# Vaccine effectiveness against symptomatic SARS-CoV-2 infection in adults aged 65 years and older in primary care: I-MOVE-COVID-19 project, Europe, December 2020 to May 2021

**DOI:** 10.2807/1560-7917.ES.2021.26.29.2100670

**Published:** 2021-07-22

**Authors:** Esther Kissling, Mariette Hooiveld, Virginia Sandonis Martín, Iván Martínez-Baz, Naoma William, Ana-Maria Vilcu, Clara Mazagatos, Lisa Domegan, Simon de Lusignan, Adam Meijer, Ausenda Machado, Mia Brytting, Itziar Casado, Josephine-L K. Murray, Sylvie Belhillil, Amparo Larrauri, Joan O’Donnell, Ruby Tsang, Marit de Lange, Ana Paula Rodrigues, Maximilian Riess, Jesús Castilla, Mark Hamilton, Alessandra Falchi, Francisco Pozo, Linda Dunford, Jade Cogdale, Tessa Jansen, Raquel Guiomar, Theresa Enkirch, Cristina Burgui, Debbie Sigerson, Thierry Blanchon, Eva María Martínez Ochoa, Jeff Connell, Joanna Ellis, Rianne van Gageldonk-Lafeber, Irina Kislaya, Angela MC Rose, Marta Valenciano, I-MOVE-COVID-19 primary care study team, I-MOVE-COVID-19 primary care study team (in addition to authors above), I-MOVE-COVID-19 primary care study team (in addition to authors above), Nick Andrews, Jamie Lopez Bernal, Heather Whitaker, Caroline Guerrisi, Titouan Launay, Shirley Masse, Sylvie van der Werf, Vincent Enouf, John Cuddihy, Adele McKenna, Michael Joyce, Cillian de Gascun, Joanne Moran, Ana Miqueleiz, Ana Navascués, Camino Trobajo-Sanmartín, Carmen Ezpeleta, Paula López Moreno, Javier Gorricho, Eva Ardanaz, Fernando Baigorria, Aurelio Barricarte, Enrique de la Cruz, Nerea Egüés, Manuel García Cenoz, Marcela Guevara, Conchi Moreno-Iribas, Carmen Sayón, Verónica Gomez, Baltazar Nunes, Rita Roquete, Adriana Silva, Aryse Melo, Inês Costa, Nuno Verdasca, Patrícia Conde, Diogo FP Marques, Anna Molesworth, Leanne Quinn, Miranda Leyton, Selin Campbell, Janine Thoulass, Jim McMenamin, Ana Martínez Mateo, Luca Basile, Daniel Castrillejo, Carmen Quiñones Rubio, Concepción Delgado-Sanz, Jesús Oliva.

**Affiliations:** 1 Epiconcept, Paris, France; 2 Nivel, Utrecht, the Netherlands; 3 National Centre for Microbiology, Institute of Health Carlos III, Madrid, Spain; 4 Instituto de Salud Pública de Navarra (IdiSNA), Pamplona, Spain; 5 Consortium for Biomedical Research in Epidemiology and Public Health (CIBERESP), Institute of Health Carlos III, Madrid, Spain; 6 Public Health Scotland, Glasgow, Scotland; 7 INSERM, Sorbonne Université, Institut Pierre Louis d'épidémiologie et de Santé Publique (IPLESP UMRS 1136), Paris, France; 8 National Centre for Epidemiology, Institute of Health Carlos III, Madrid, Spain; 9 Health Service Executive-Health Protection Surveillance Centre, Dublin, Ireland; 10 Nuffield Department of Primary Care Health Sciences, University of Oxford, Oxford, UK; 11 Royal College of General Practitioners Research and Surveillance Centre, London, UK; 12 National Institute for Public Health and the Environment (RIVM), Bilthoven, the Netherlands; 13 Instituto Nacional de Saúde Dr. Ricardo Jorge, Lisbon, Portugal; 14 The Public Health Agency of Sweden, Stockholm, Sweden; 15 Unité de Génétique Moléculaire des Virus à ARN, UMR 3569 CNRS, Université Paris Diderot SPC, Institut Pasteur, Paris, France; 16 CNR des virus des infections respiratoires, Institut Pasteur, Paris, France; 17 Laboratoire de Virologie, Université de Corse-Inserm, Corte, France; 18 National Virus Reference Laboratory, University College Dublin, Dublin, Ireland; 19 Public Health England, London, England; 20 Servicio de Epidemiología y Prevención Sanitaria, Dirección General de Salud Pública, Consumo y Cuidados, La Rioja, Spain; 21 The members of the I-MOVE-COVID-19 primary care study team are listed in the Investigators tab

**Keywords:** COVID-19, SARS-CoV-2, vaccine effectiveness, multicentre study, test-negative design, Europe

## Abstract

We measured COVID-19 vaccine effectiveness (VE) against symptomatic SARS-CoV-2 infection at primary care/outpatient level among adults ≥ 65 years old using a multicentre test-negative design in eight European countries. We included 592 SARS-CoV-2 cases and 4,372 test-negative controls in the main analysis. The VE was 62% (95% CI: 45–74) for one dose only and 89% (95% CI: 79–94) for complete vaccination. COVID-19 vaccines provide good protection against COVID-19 presentation at primary care/outpatient level, particularly among fully vaccinated individuals.

The I-MOVE-COVID-19 network collates epidemiological and clinical information on patients with coronavirus disease (COVID-19), including severe acute respiratory syndrome coronavirus 2 (SARS-CoV-2) virological characterisation in 11 European countries [1]. One component of I-MOVE-COVID-19 is the multicentre vaccine effectiveness (VE) study at primary care/outpatient level in nine European study sites in eight countries. We measured overall and product-specific COVID-19 VE against symptomatic SARS-CoV-2 infection among those aged 65 years and older. We also measured VE by time since vaccination.

## Participating study sites

The I-MOVE-COVID-19 primary care/outpatient study sites are: England, France, Ireland, the Netherlands, Portugal, Scotland, Spain (three regions), Spain (Navarra region) and Sweden (Table 1). Study sites adapted the I-MOVE-COVID-19 VE study protocol [2], to their country-specific setting. Sites included all, or a systematic sample of, acute respiratory infection (ARI) patients or patients presenting with common COVID-19 symptoms (at least one of the following symptoms: fever, cough, shortness of breath, sudden onset of anosmia/ageusia) who contacted the sentinel physician (Table 1). The Navarra region included all patients presenting to the health service primary care physicians. A systematic sample of patients in COVID-19 testing centres both with and without physician referral were included in Portugal. Scotland included a systematic sample of patients presenting to community-based COVID-19 centres with self- and clinical staff-based swabbing.

**Table 1:** Characteristics of study sites participating in the primary care/outpatient I-MOVE-COVID-19 vaccine effectiveness study, Europe, December 2020–May 2021 (n = 9)

Site	Comprehensive /sentinel	Primary care physician (PC) / community (Com)	Swab collection	Vaccines used in study population^a^ Cominarty (C), Vaxzevria (V), Spikevax (S), Janssen (J)	Source of information Electronic medical records (E), vaccine registry (VR), GP interview (GPI), patient questionnaire (P), other health databases, including national laboratory databases (H)	Laboratory tests used RT-PCR (PCR), rapid antigen test (RAT)
**England**	Sentinel	PC	At GP practice	C, V	E, VR	PCR
**France**	Sentinel	PC	At medical laboratory or at GP practice	C, V, S, J	GPI	PCR, RAT
**Ireland**	Sentinel	PC	At COVID-19 testing centre	C, V, S	VR, GP, H	PCR
**Netherlands**	Sentinel	PC	At GP practice	Information on vaccine type not collected	GPI	PCR
**Portugal**	Sentinel	PC	At COVID-19 testing centre	C, V, S	GPI, H, VR, E	PCR, RAT
**Scotland**	Sentinel	Com	At COVID-19 testing centre	C, V	P	PCR
**Spain (three regions)**	Sentinel	PC	At GP practice	C, V	GPI, E, VR	PCR, RAT
**Spain (Navarra)**	Comprehensive	PC	At GP practice	C, V, S, J	E, VR	PCR, RAT
**Sweden**	Sentinel	PC	At GP practice	C	GPI, H, VR	PCR

C: Cominarty (BNT162b2, BioNTech-Pfizer, Mainz, Germany/New York, United States); Com: community; COVID-19: coronavirus disease; E: electronic medical records; GP: general practice; GPI: general practitioner interview; H: other health databases, including national laboratory databases; J: Janssen (Ad26.COV2-S (recombinant), Janssen-Cilag International NV, Beerse, Belgium); P: patient questionnaire; PC: primary care physician; RT-PCR: real time reverse transcription–polymerase chain reaction; RAT: rapid antigen test; S: Spikevax (mRNA-1273, Moderna, Cambridge, US), US; V: Vaxzevria (ChAdOx1/nCoV-19, AstraZeneca, Cambridge, United Kingdom); VE: vaccine effectiveness; VR: vaccine registry.

^a^ Study population between 10 December 2020 and 31 May 2021. Other vaccines may have been used in these countries, but were not captured within this study.

Demographic and clinical information including age, sex, any underlying chronic conditions and conditions relevant to COVID-19 (diabetes, heart disease, chronic lung disease, immunodeficiencies), and COVID-19 vaccination status was collected (Table 1).

## Definitions of cases and controls and vaccination

We used the test-negative design [3], where cases were patients testing SARS-CoV-2 positive and controls were patients testing SARS-CoV-2 negative. We included patients aged 65 years and older belonging to the COVID-19 vaccine age-specific target groups at time of swab collection, between 10 December 2020 and 31 May 2021. We excluded patients with missing age, patients in residential care homes, patients previously testing positive for SARS-CoV-2 (where information was known/reported), controls testing positive for seasonal coronaviruses and patients with missing COVID-19 vaccination status and/or date of vaccination. We included only those who were swabbed within 10 days (for investigation with RT-PCR) or 5 days (for investigation with rapid antigen tests) of symptom onset. In study sites where date of symptom onset was not available (one site) or had ≥ 25% of missing information (two sites), we imputed it as 3 days before the swab date, as 3 days was the median delay between onset and swab in the pooled data.

We defined a person as having received ‘at least one dose of vaccine’ if received at least one dose of vaccine ≥ 14 days before symptom onset, vaccinated with ‘one dose only’ if received only one of two recommended doses ≥ 14 days before symptom onset, and a person ‘completely vaccinated’ if received one dose of Janssen vaccine or the second dose of other vaccines ≥ 14 days before symptom onset.

Persons were considered to be ‘unvaccinated’ if they had not received any vaccine or were vaccinated on the day that symptoms started or after symptom onset. Those receiving their first dose of vaccine < 14 days before symptom onset were excluded from the main analysis for VE of at least one dose or VE of one dose only. Those receiving their first dose of Janssen vaccine or second dose of other vaccines < 14 days before symptom onset were excluded from the VE analysis for complete vaccination. These persons were included in the analysis of VE by time since vaccination for vaccination either 1–4 days or 6–13 days before symptom onset.

In five study sites where technically feasible, the whole or partial genome of all or a random selection of viruses confirmed by PCR was sequenced. Two further study sites sequence viruses from confirmed PCR cases, but selection for sequencing was not random throughout the study period. Phylogenetic analysis was performed to identify the Phylogenetic Assignment of Named Global Outbreak (Pango) lineage based on the classification of 15 June 2021.

## Statistical analysis

We compared the odds of vaccination between cases and controls. We computed VE as 1 minus the OR, expressed as a percentage. We used logistic regression to model the OR, including study site as a fixed effect and month of swab taken in a crude analysis. In addition, in adjusted analyses, we included age group, sex and presence of at least one underlying chronic condition relevant to COVID-19 (diabetes, heart disease, chronic lung disease, immunodeficiencies).

We also conducted a secondary analysis excluding Scotland in view of differences with other protocols (see Supplementary Material), and sensitivity analyses excluding one study site with ca 25% of missing PCR/RAT test results and study sites with imputed dates of onset. In further sensitivity analyses, we varied the imputed onset dates between 2 and 5 days before swab date. We measured VE up to the end of April 2021 to assess the effect of high vaccine coverage rates in older age groups as time progressed.

To avoid small sample bias, we did not present any analyses where there were five or fewer vaccinated cases or controls.

### Ethical statement

The planning, conduct and reporting of the studies was in line with the Declaration of Helsinki. Official ethical approval was not required if studies were classified as part of routine care/surveillance (i.e. in the Netherlands, Spain (three regions), England, Scotland and Ireland). Other study sites obtained local ethical approval from a national review board. This was the case for France (French Data Protection Agency (registration number CNIL#471393) and the French ethics research committee (‘Comité de Protection des Personnes’ – CPP)); Navarre (registration number PI2020/45); Portugal (Ethics Committee of Instituto Nacional de Saúde Doutor Ricardo Jorge, no registration number given) and Sweden (registration number 2006/1040–31/2).

## Vaccine effectiveness results

### Main analysis

In the main analysis, we included 4,964 patients (Supplementary Figure S1), comprising 592 cases and 4,372 controls (Supplementary Figure S2).

Among them, 51% of cases (299/592) and 43% of controls (1,878/4,372) were aged 65–74 years (Supplementary Table S1). Among cases, 14% (84/592) were vaccinated with at least one dose and 9% (52/592) were vaccinated with one dose only of COVID-19 vaccine. Among those vaccinated with at least one dose, where vaccine product was known, 73% (61/83) were vaccinated with Comirnaty. Among controls, 46% (2,014/4,372) were vaccinated with at least one dose of COVID-19 vaccine and 20% (866/4,368) were vaccinated with one dose only of COVID-19 vaccine. Among those vaccinated with at least one dose, where vaccine product was known, 66% (1,327/1,998) were vaccinated with the Comirnaty vaccine.

There were 465 cases presenting in the seven study sites undertaking sequencing. Of those, 31 (7%) were sequenced and 27 (87%) of these belonged to the SARS-CoV-2 Alpha variant (B.1.1.7) of concern (Supplementary Table S2).

The adjusted VE for any vaccine for one dose only and complete vaccination against symptomatic infection were 62% (95% CI: 45–74) and 89% (95% CI: 79–94), respectively (Table 2). The VE for Comirnaty for one dose only and complete vaccination were 61% (95% CI: 39–75) and 87% (95% CI: 74–93), respectively. The VE for Vaxzevria for one dose only was 68% (95% CI: 39–83). We could not estimate VE for Vaxzevria for completed vaccination and any VE for Janssen and Spikevax due to small sample size.

**Table 2:** Effectiveness of COVID-19 vaccination among participants aged 65 years and older in the primary care/outpatient I-MOVE-COVID-19 vaccine effectiveness study, Europe, December 2020–May 2021

Main analysis (Nine study sites)			
**Analysis type and vaccination status**	**Cases; vaccinated/ controls; vaccinated**	**Crude VE (95% CI)^a^**	**Adjusted VE (95% CI)^b^**
At least one dose^c^	592; 84 / 4,372; 2,014	74 (64–80)	73 (63–81)
One dose only^d^	560; 52 / 3,224; 866	62 (45–74)	62 (45–74)
Completely vaccinated^e^	522; 14 / 3037; 679	88 (79–94)	89 (79–94)
**Comirnaty (BNT162b2, BioNTech-Pfizer, Mainz, Germany/New York, United States) vaccine**			
At least one dose^c^	566; 61 / 3,672; 1327	73 (62–80)	74 (63–82)
One dose only^d^	535; 30 / 2,778; 433	60 (38–74)	61 (39–75)
Completely vaccinated^e^	519; 14 / 2,857; 512	85 (73–92)	87 (74–93)
**Vaxzevria (ChAdOx1/nCoV-19, AstraZeneca, Cambridge, United Kingdom) vaccine**			
At least one dose^c^	523; 18 / 2,810; 465	75 (52–87)	74 (50–86)
One dose only^d^	523; 18 / 2,687; 342	68 (41–83)	68 (39–83)
Completely vaccinated^e^	Sample size too small	NA	NA

CI: confidence interval; COVID-19: coronavirus disease; NA: not applicable; VE: vaccine effectiveness.

^a^ Adjusted by study site, month of swab taken (due to low numbers December and January were grouped as one category).

^b^ Adjusted by study site, month of swab taken (due to low numbers December and January were grouped as one category), 10-year age group, sex. Due to substantial missing data on chronic conditions from one study site, the estimates were not adjusted by chronic condition.

^c^ First dose of any COVID-19 vaccine received ≥ 14 days before onset.

^d^ One of two recommended doses of COVID-19 vaccine received ≥ 14 days before onset. Excludes Janssen vaccine as it is a 1-dose vaccine.

^e^ Second dose of COVID-19 vaccine received ≥ 14 days before onset, or first dose received ≥ 14 days before onset if Janssen vaccine. Eighteen cases and 465 controls received a second dose of any vaccine <14 days before onset. In the Comirnaty-specific analysis, 17 cases and 381 controls received a second dose of vaccine <14 days before onset.

The adjusted odds for having received one dose of any 2-dose COVID-19 vaccine was 14% greater among controls than among cases in 1–4 days between vaccination and onset and 12% and 30% for the Comirnaty and Vaxzevria vaccines, respectively (Table 3).

**Table 3:** Odds ratio of COVID-19 vaccination by time since vaccination to symptom onset among participants receiving one dose only of 2-dose COVID-19 vaccines in the primary care/outpatient I-MOVE-COVID-19 vaccine effectiveness study, Europe, December 2020–May 2021

Main analysis (nine study sites)			
**Analysis type and one dose vaccination status**	**Cases / controls**	**OR ** **(95% CI)^a^**	**AOR (95% CI)^b^**
**Any COVID-19 vaccine (except Janssen (Ad26.COV2-S (recombinant), Janssen-Cilag International NV, Beerse, Belgium)**			
Unvaccinated	508/2,358	Ref	Ref
1–4 days^c^	36/243	0.89 (0.60–1.31)	0.86 (0.58–1.27)
5–13 days^d^	59/503	0.64 (0.47–0.89)	0.63 (0.45–0.87)
≥ 14 days^e^	52/866	0.38 (0.26–0.55)	0.38 (0.26–0.55)
**Comirnaty (BNT162b2, BioNTech-Pfizer, Mainz, Germany/New York, United States) vaccine**			
Unvaccinated	506/2,352	Ref	Ref
1–4 days^c^	27/169	0.92 (0.59–1.43)	0.88 (0.56–1.39)
5–13 days^d^	50/366	0.70 (0.50–0.99)	0.68 (0.48–0.97)
≥ 14 days^e^	30/433	0.40 (0.26–0.62)	0.39 (0.25–0.61)
**Vaxzevria (ChAdOx1/nCoV-19, AstraZeneca, Cambridge, United Kingdom) vaccine**			
Unvaccinated	505/2,345	Ref	Ref
1–4 days^c^	6/56	0.71 (0.29–1.74)	0.70 (0.28–1.73)
5–13 days^d^	3/102	Sample size too small	Sample size too small
≥ 14 days^e^	18/342	0.32 (0.17–0.59)	0.32 (0.17–0.61)

AOR: adjusted odds ratio; CI: confidence interval; COVID-19: coronavirus disease; OR: odds ratio; Ref: reference; VE: vaccine effectiveness.

^a^ Adjusted by study site, month of swab (due to low numbers December and January were one category).

^b^ Adjusted by study site, month of swab (due to low numbers December and January were one category), 10-year age group, sex. Due to substantial missing data on chronic conditions from one study site, the estimates were not adjusted by chronic condition.

^c^ First dose of 2-dose COVID-19 vaccine received 1–4 days before onset, patients with unknown doses or two doses excluded.

^d^ First dose of 2-dose COVID-19 vaccine received 5–13 days before onset, patients with unknown doses or two doses excluded.

^e^ First dose of 2-dose COVID-19 vaccine received ≥ 14 days before onset, patients with unknown doses or two doses excluded.

### Sensitivity analyses

Descriptive analyses and VE estimates of the secondary analysis with eight study sites are reported in Supplementary Tables S3, S4 and S5. The VE point estimate of one dose only of any COVID-19 vaccine was 6% lower compared with the main analysis, while the VE point estimate of complete vaccination was 2% lower.

Sensitivity analyses excluding specific study sites with different imputations for onset date and with a study period up to the end of April 2021 resulted in VE point estimates varying within 4% of the primary VE estimates (Supplementary table S6).

## Discussion

Our results suggest that vaccination with one only dose offered moderate protection (62%) against symptomatic SARS-CoV-2 infection among adults aged 65 years and older consulting at primary care level, and good protection (89%) among those who were completely vaccinated. Both the Comirnaty and Vaxzevria vaccines provide protection after one dose and good protection after two doses of Comirnaty. Sample size was too low to evaluate two doses of Vaxzevria.

These results are consistent with reported VE estimates against symptomatic COVID-19 infection among older adults in community/outpatient settings, of 47–70% [4-6] among those vaccinated with one dose and 85–96% [4-7] among fully vaccinated. Results are slightly higher compared with a community-based study among close contacts in Navarra, Spain, where VE point estimates against symptomatic COVID-19 infection among those aged 60 years and older were 30% (95% CI: 10–45) among partially vaccinated and 77% (95% CI: 56–88) among fully vaccinated [8]. Differences could be explained by a multitude of factors, such as characteristics of the study population, case definitions, differential virus exposures, vaccination status definitions (including use of swab date instead of date of symptom onset), vaccines used, circulating variants and random variation.

We observed some effect from the vaccine (14%) among those vaccinated with one dose 1–4 days before symptom onset when no effect of the vaccine should be evident, although confidence intervals include 0%. This has also been observed by Lopez Bernal et al. [4], where a greater effect for the Vaxzevria vaccine compared with the Comirnaty vaccine was observed. This effect could be explained by an increase in SARS-CoV-2 testing among vaccinated controls, due to onset of systemic symptoms caused by the vaccine for a few days after vaccination [9]. Alternatively, persons with COVID-19 symptoms or contacts of cases may defer vaccination. We observed some vaccine effect (32%) among those vaccinated 5–13 days before symptom onset. This may be in part due to the imputation of onset date in three sites, which potentially led to the inclusion of persons vaccinated ≥ 14 days before symptom onset in this interval, where a vaccine effect is expected, or other unmeasured confounding, and warrants further investigation.

This is a multicentre study among settings with different patient pathways in terms of location of swabbing, vaccines used, sources of information and laboratory investigations performed. When we excluded one study site with purely community-based study including self-collected swabs, this changed the VE of any COVID-19 vaccine by ≤ 6%.

Limitations include small sample sizes in some of the study sites. However, a multicentre design to measure VE in the context of different vaccines used and vaccine schedules can be a strength. We could not measure variant-specific VE due to the small proportion of sequencing results. Measuring variant-specific VE is an aim of our group in the near future as sequencing capacity increases.

As vaccination rolls out, certain target groups for vaccination are becoming highly vaccinated, e.g., at time of writing the proportions of those aged 80 years and over vaccinated with one and two doses were approaching 95–100% in some countries [10]. Those unvaccinated in this target group may be an unusual population, with potentially a different risk of infection or exposure to the virus, thus violating core criteria of VE studies [11].

To check for this potential bias, we measured VE up to the end of April and the results were the same compared with the VE up to the end of May (Supplementary Table S6). Future studies need to take this challenge into account.

In three sites where previous SARS-CoV-2 infection was documented, we excluded controls with previous infection. However, even in those sites, previous infection is not always known, particularly with asymptomatic infections, and persons with previous infection could have been included among all study sites. This may have introduced bias in our results if previous infection influenced vaccination status.

In the main analysis, VE was not adjusted by presence of chronic condition, due to a large amount of missing data from one study site. In the secondary analysis, results differed only by ≤ 1% after adjusting by presence of chronic condition, indicating that chronic condition may not be a confounder in this study population. In all analyses month of swab and age were the strongest confounders.

This is an observational study with potential further selection and information biases than those mentioned above. However, the test-negative design used, reduces bias due to healthcare seeking behaviour [3,12-14].

## Conclusions

COVID-19 vaccination provides substantial protection against COVID-19 presentation at primary care/outpatient level among those aged 65 and older, particularly after completed vaccination. As the vaccines do not provide 100% protection, vaccinated persons should continue to follow public health guidance, including hygiene and physical distancing measures. Next steps for the I-MOVE-COVID-19 VE study are to include more in-depth analysis of age and risk groups, VE by time since vaccination and increasing sequencing data of cases to measure variant-specific VE.

## I-MOVE-COVID-19 primary care study team (in addition to authors above)

Nick Andrews, Public Health England, London, United Kingdom

Jamie Lopez Bernal, Public Health England, London, United Kingdom

Heather Whitaker, Public Health England, London, United Kingdom

Caroline Guerrisi - Sorbonne Université, INSERM, Institut Pierre Louis d'épidémiologie et de Santé Publique (IPLESP UMRS 1136), Paris, France

Titouan Launay - Sorbonne Université, INSERM, Institut Pierre Louis d'épidémiologie et de Santé Publique (IPLESP UMRS 1136), Paris, France

Shirley Masse - Laboratoire de Virologie, Université de Corse-Inserm, Corte, France

Sylvie van der Werf, Unité de Génétique Moléculaire des Virus à ARN, UMR 3569 CNRS, Université Paris Diderot SPC, Institut Pasteur, Paris; CNR des virus des infections respiratoires, Institut Pasteur, Paris, France

Vincent Enouf, Unité de Génétique Moléculaire des Virus à ARN, UMR 3569 CNRS, Université Paris Diderot SPC, Institut Pasteur, Paris; CNR des virus des infections respiratoires, Institut Pasteur, Paris, France

John Cuddihy, Health Service Executive-Health Protection Surveillance Centre, Dublin, Ireland

Adele McKenna, Health Service Executive-Health Protection Surveillance Centre, Dublin, Ireland

Michael Joyce, Irish College of General Practitioners, Dublin, Ireland

Cillian de Gascun, National Virus Reference Laboratory, University College Dublin, Dublin, Ireland

Joanne Moran, National Virus Reference Laboratory, University College Dublin, Dublin, Ireland

Ana Miqueleiz, Complejo Hospitalario de Navarra, Pamplona, Spain

Ana Navascués, Complejo Hospitalario de Navarra, Pamplona, Spain

Camino Trobajo-Sanmartín, Complejo Hospitalario de Navarra, Pamplona, Spain

Carmen Ezpeleta, Complejo Hospitalario de Navarra, Pamplona, Spain

Paula López Moreno, Servicio Navarro de Salud-Osasunbidea, Pamplona, Spain

Javier Gorricho, Servicio Navarro de Salud-Osasunbidea, Pamplona, Spain

Eva Ardanaz, Instituto de Salud Pública y Laboral de Navarra—IdiSNA—CIBERESP, Pamplona, Spain

Fernando Baigorria, Instituto de Salud Pública y Laboral de Navarra—IdiSNA—CIBERESP, Pamplona, Spain Aurelio Barricarte, Instituto de Salud Pública y Laboral de Navarra—IdiSNA—CIBERESP, Pamplona, Spain

Enrique de la Cruz, Instituto de Salud Pública y Laboral de Navarra—IdiSNA—CIBERESP, Pamplona, Spain

Nerea Egüés, Instituto de Salud Pública y Laboral de Navarra—IdiSNA—CIBERESP, Pamplona, Spain

Manuel García Cenoz, Instituto de Salud Pública y Laboral de Navarra—IdiSNA—CIBERESP, Pamplona, Spain

Marcela Guevara, Instituto de Salud Pública y Laboral de Navarra—IdiSNA—CIBERESP, Pamplona, Spain

Conchi Moreno-Iribas, Instituto de Salud Pública y Laboral de Navarra—IdiSNA—CIBERESP, Pamplona, Spain

Carmen Sayón, Instituto de Salud Pública y Laboral de Navarra—IdiSNA—CIBERESP, Pamplona, Spain

Verónica Gomez, Departamento de Epidemiologia, Instituto Nacional de Saúde Dr. Ricardo Jorge, Lisbon, Portugal

Baltazar Nunes, Departamento de Epidemiologia, Instituto Nacional de Saúde Dr. Ricardo Jorge, Lisbon, Portugal

Rita Roquete, Departamento de Epidemiologia, Instituto Nacional de Saúde Dr. Ricardo Jorge, Lisbon, Portugal

Adriana Silva, Departamento de Epidemiologia, Instituto Nacional de Saúde Dr. Ricardo Jorge, Lisbon, Portugal

Aryse Melo, Departamento de Doenças Infeciosas, Instituto Nacional de Saúde Dr. Ricardo Jorge, Lisbon, Portugal

Inês Costa, Departamento de Doenças Infeciosas, Instituto Nacional de Saúde Dr. Ricardo Jorge, Lisbon, Portugal

Nuno Verdasca, Departamento de Doenças Infeciosas, Instituto Nacional de Saúde Dr. Ricardo Jorge, Lisbon, Portugal

Patrícia Conde, Departamento de Doenças Infeciosas, Instituto Nacional de Saúde Dr. Ricardo Jorge, Lisbon, Portugal

Diogo FP Marques, Public health Scotland, Glasgow, United Kingdom

Anna Molesworth, Public health Scotland, Glasgow, United Kingdom

Leanne Quinn, Public health Scotland, Glasgow, United Kingdom

Miranda Leyton, Public health Scotland, Glasgow, United Kingdom

Selin Campbell, Public health Scotland, Glasgow, United Kingdom

Janine Thoulass, Public health Scotland, Glasgow, United Kingdom

Jim McMenamin, Public health Scotland, Glasgow, United Kingdom

Ana Martínez Mateo, Subdirección General de Vigilancia y Respuesta a Emergencias de Salud Pública, Agencia de Salud Pública, Catalunya and CIBER Epidemiologia y Salud Pública (CIBERESP), Barcelona, Spain

Luca Basile, Subdirección General de Vigilancia y Respuesta a Emergencias de Salud Pública, Agencia de Salud Pública, Catalunya and CIBER Epidemiologia y Salud Pública (CIBERESP), Barcelona, Spain

Daniel Castrillejo, Servicio de Epidemiología, Dirección General de Salud Pública y Consumo, Melilla, Spain

Carmen Quiñones Rubio, Servicio de Epidemiología y Prevención Sanitaria, Dirección General de Salud Pública, Consumo y Cuidados, La Rioja, Spain

Concepción Delgado-Sanz, National Centre for Epidemiology, Institute of Health Carlos III, Madrid, Spain, Consortium for Biomedical Research in Epidemiology and Public Health (CIBERESP), Institute of Health Carlos III, Madrid, Spain

Jesús Oliva, National Centre for Epidemiology, Institute of Health Carlos III, Madrid, Spain, Consortium for Biomedical Research in Epidemiology and Public Health (CIBERESP), Institute of Health Carlos III, Madrid, Spain

## Supplementary Data

1005000 Supplementary Material
