# Supplementary material for: Vaccine effectiveness against symptomatic SARS-CoV-2 infection in adults aged 65 years and older in primary care: I-MOVE-COVID-19 project, Europe, December 2020 to May 2021
Source: Euro Surveill. 2021 Jul 22;26(29):2100670. doi: 10.2807/1560-7917.ES.2021.26.29.2100670 (PMC8299744; doi:10.2807/1560-7917.ES.2021.26.29.2100670)
Supplement: Supplementary Material [file 21-00670_KISSLING_Supplementary_material.pdf]

Supplementary material

This supplementary material is hosted by *Eurosurveillance* as supporting information alongside the article “Vaccine effectiveness against symptomatic SARS-CoV-2 infection in adults aged 65 years and older in primary care: I-MOVE-COVID-19 project, Europe, December 2020 to May 2021” on behalf of the authors who remain responsible for the accuracy and appropriateness of the content. The same standards for ethics, copyright, attributions and permissions as for the article apply. Supplements are not edited by *Eurosurveillance* and the journal is not responsible for the maintenance of any links or email addresses provided therein.

Supplementary Figure S1: Flowchart of exclusions for participants in the primary care/outpatient I-MOVE-COVID-19 VE study, Europe, December 2020–May 2021

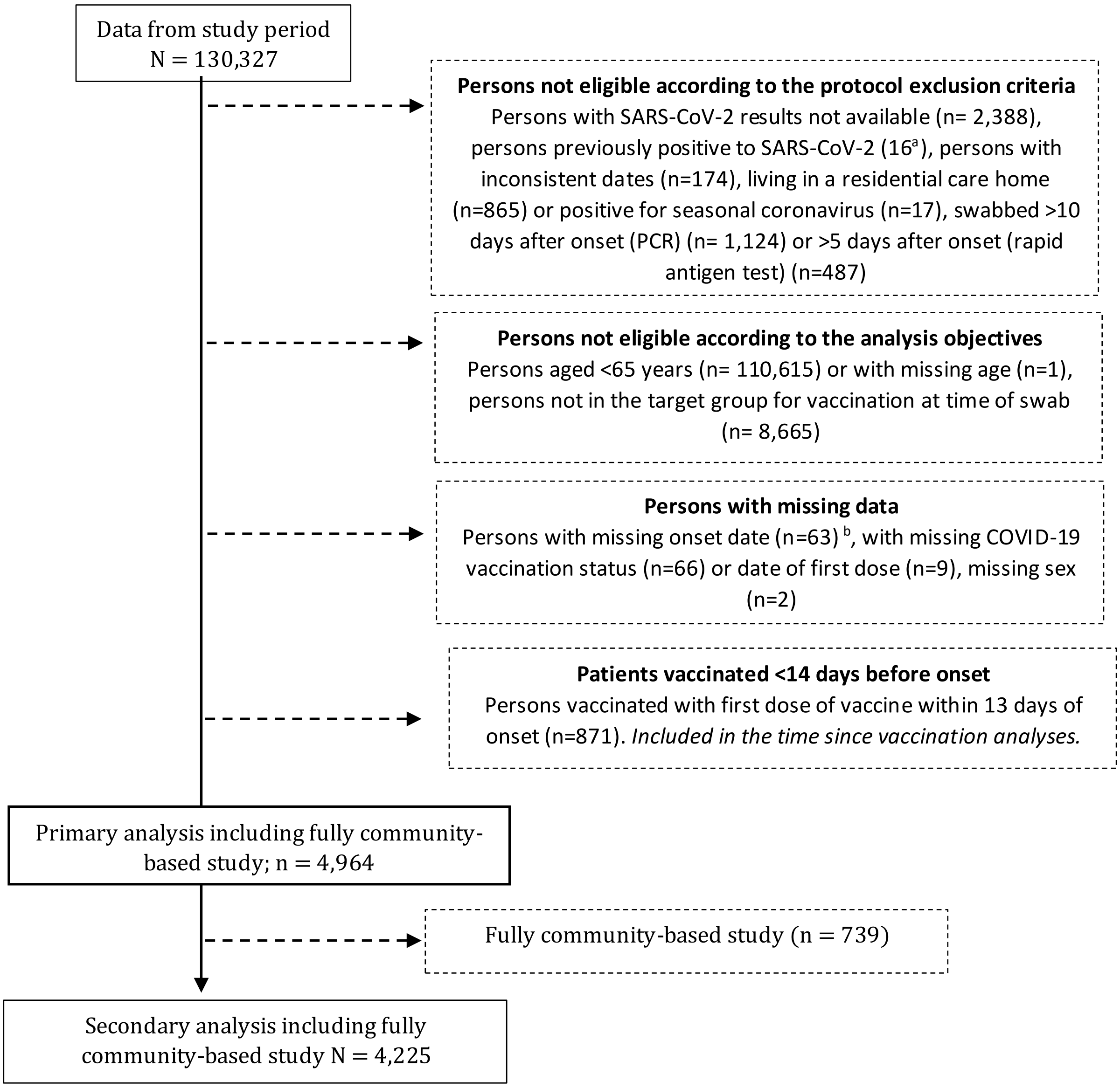

a In NA these patients were excluded prior to sending to the central hub and not included in the flowchart.

b Onset dates were imputed in NA, IE, PT.

Main analysis

Supplementary Figure S2: Number of persons by week of swab and case status, number of persons vaccinated with first dose by week of vaccination in the primary care/outpatient I-MOVE-COVID-19 VE study, Europe, December 2020–May 2021

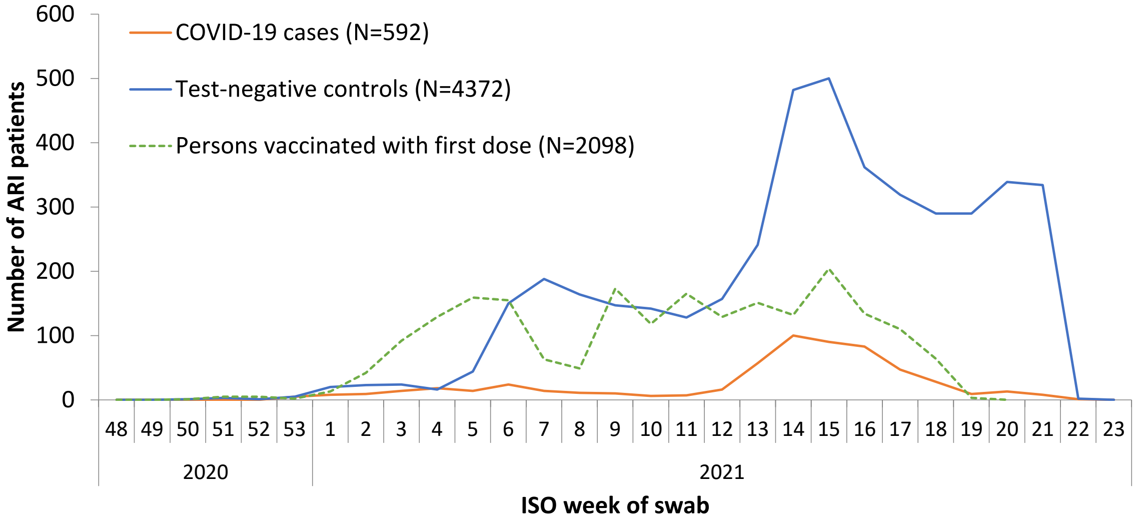

Supplementary Table S1. Descriptive analysis of participants in the primary care/outpatient I-MOVE-COVID-19 VE study main analysis, Europe (9 study sites), December 2020–May 2021

Characteristic	SARS-CoV-2 cases (n=592)		Test-negative controls (n=4372)	
	Number	%	Number	%
**Age group**				
65-74	299	51	1878	43
75-84	176	30	1468	34
85+	117	20	1026	23
**Sex**				
Female	331	56	2604	60
Male	261	44	1768	40
**Presence of chronic condition**[a]				
Presence of chronic condition	284	50	2074	54
No chronic condition	279	50	1734	46
Missing	29		564	
**COVID-19 vaccination status**				
Unvaccinated	508	86	2358	54
Vaccinated with at least one dose[b]	84	14	2014	46
**COVID-19 vaccination status**				
Unvaccinated	508	86	2358	54
One dose only[c]	52	9	866	20
Complete vaccination[d]	14	2	679	16

Characteristic	SARS-CoV-2 cases (n=592)		Test-negative controls (n=4372)	
	Number	%	Number	%
Vaccinated with the second dose or first dose of Janssen vaccine <14 days before onset[e]	18	3	465	11
Vaccination date/status of second dose missing	0		4	
**Vaccine brand of first dose among vaccinated**				
Vaxzevria (AstraZeneca)	18	22	465	23
Spikevax (Moderna)	4	5	179	9
Comirnaty (Pfizer/BioNTech)	61	73	1327	66
Janssen	0	0	27	1
Unknown	1		16	
**Month of swab**				
December 2020	0	0	5	0
January 2021	54	9	88	2
February 2021	63	11	546	12
March 2021	55	9	651	15
April 2021	356	60	1794	41
May 2021	64	11	1288	29
**Study site**				
EN	13	2	58	1
ES	80	14	32	1
FR	84	14	69	2
IE	4	1	88	2
NA	363	61	3397	78
NL	3	1	8	0
PT	1	0	14	0
SC	43	7	696	16
SE	1	0	10	0

[a] Among those commonly collected: diabetes, heart disease, chronic lung disease, immunodeficiencies; For IE: Any medical risk condition.

[b] First dose of any COVID-19 vaccine received ≥14 days before onset.

[c] One of two recommended doses of COVID-19 vaccine received ≥14 days before onset. Excludes Janssen vaccine.

[d] Second dose of COVID-19 vaccine received ≥14 days before onset, or first dose received ≥14 days before onset if Janssen vaccine.

[e] Second dose of COVID-19 vaccine received <14 days before onset, or first dose received <14 days before onset if Janssen vaccine. These persons are excluded in the analysis of "complete vaccination".

Supplementary Table S2. SARS-CoV-2 viruses characterised by variant of concern, among study sites included in the VE analysis reporting lineage (7 study sites), I-MOVE-COVID-19 VE study analysis, Europe, December 2020–May 2021

**VOC (Pango lineage)**	**n**
Total SARS-CoV-2 among those sites with lineage information among study participants (n=7 sites)	n = 465
Sequenced SARS-CoV-2 viruses[a]	31
Alpha (B.1.1.7)	*27*
Beta (B.1.351)	*0*
Gamma (P.1)	*0*
Delta (B.1.617.2)	*0*
No VOC	*4*

[a] 7% (31/465) sequenced overall. 17% (31/184) sequenced among those tested with RT-PCR.

Secondary analysis

Supplementary Table S3. Descriptive analysis of participants in the primary care/outpatient I-MOVE-COVID-19 VE study secondary analysis, Europe (8 study sites[a]), December 2020–May 2021

Characteristic	SARS-CoV-2 cases (n=549)		Test-negative controls (n=3676)	
	Number	%	Number	%
**Age group**				
65-74	286	52	1549	42
75-84	159	29	1215	33
85+	104	19	912	25
**Sex**				
Female	308	56	2183	59
Male	241	44	1493	41
**Presence of chronic condition**[b]				
Presence of chronic condition	269	49	1948	53
No chronic condition	280	51	1728	47
Missing	3		2	
**COVID-19 vaccination status**				
Unvaccinated	477	87	2215	60
Vaccinated with at least one dose[c]	72	13	1461	40
**COVID-19 vaccination status**				
Unvaccinated	477	87	2215	60
One dose only[d]	41	7	482	13
Complete vaccination[e]	14	3	570	16
Vaccinated with the second dose or first dose of Janssen vaccine <14 days before onset[f]	17	3	405	11
Vaccination date/status of second dose missing	0		4	
**Vaccine brand of first dose among vaccinated**				
Vaxzevria (AstraZeneca)	9	13	90	6
Spikevax (Moderna)	4	6	179	12
Comirnaty (Pfizer/BioNTech)	58	82	1149	79
Janssen	0	0	27	2
Unknown	1		16	
**Month of swab**				
December 2020	0	0	5	0
January 2021	82	6	447	1
February 2021	104	9	876	11
March 2021	404	10	2064	12
April 2021	413	64	2768	44
May 2021	63	11	1160	32
**Study site**				

Characteristic	SARS-CoV-2 cases (n=549)		Test-negative controls (n=3676)	
	**Number**	**%**	**Number**	**%**
EN	13	2	58	2
ES	80	14	32	1
FR	84	15	69	2
IE	4	1	88	2
NA	363	66	3397	92
NL	3	1	8	0
PT	1	0	14	0
SE	1	0	10	0

[a] The secondary analysis excludes Scotland, a study site that is exclusively community-based and includes self-swabbing.

[b] Among those commonly collected: diabetes, heart disease, chronic lung disease, immunodeficiencies; For IE: Any medical risk condition.

[c] First dose of any COVID-19 vaccine received ≥14 days before onset.

[d] One of two recommended doses of COVID-19 vaccine received ≥14 days before onset. Excludes Janssen vaccine.

[e] Second dose of COVID-19 vaccine received ≥14 days before onset, or first dose received ≥14 days before onset if Janssen vaccine.

[f] Second dose of COVID-19 vaccine received <14 days before onset, or first dose received <14 days before onset if Janssen vaccine. These persons are excluded in the analysis of “complete vaccination”.

Supplementary Table S4. Effectiveness of COVID-19 vaccination among participants in the primary care/outpatient I-MOVE-COVID-19 VE study (8 study sites[a]), Europe, December 2020–May 2021

**Secondary analysis (8 study sites[a])**				
**Analysis type and vaccination status**	**Cases; vaccinated/ controls; vaccinated**	**Crude VE (95% CI)[b]**	**Adjusted VE (95% CI)[c]**	**Adjusted VE (95% CI)[d]**
At least one dose[e]	546;71 / 3674;1460	72 (61 to 79)	72 (60 to 80)	72 (60 to 80)
One dose only[f]	515;40 / 2696;482	56 (34 to 70)	56 (34 to 71)	56 (34 to 70)
Completely vaccinated[g]	489;14 / 2783;569	86 (75 to 92)	87 (75 to 93)	87 (75 to 93)
**Comirnaty vaccine**				
At least one dose[e]	530;58 / 3349;1148	70 (58 to 79)	71 (58 to 80)	71 (57 to 80)
One dose only[f]	500;28 / 2504;303	48 (19 to 67)	49 (19 to 68)	48 (17 to 68)
Completely vaccinated[g]	486;14 / 2683;482	84 (71 to 91)	86 (72 to 92)	85 (72 to 92)
**Vaxzevria vaccine**				
At least one dose[e]	480;8 / 2291;90	58 (0 to 82)	57 (-3 to 82)	56 (-6 to 82)
One dose only[f]	480;8 / 2289;88	57 (-2 to 82)	56 (-6 to 82)	55 (-9 to 81)
Completely vaccinated[g]	Sample size too small			

CI: confidence interval; VE: vaccine effectiveness.

[a] The secondary analysis excludes Scotlan, a study site that is exclusively community-based and includes self-swabbing.
[b] Adjusted by study site, month of swab taken (due to low numbers December and January were grouped as one category).
[c] Adjusted by study site, month of swab taken (due to low numbers December and January were grouped as one category), 10-year age group, sex.
[d] Adjusted by study site, month of swab (due to low numbers December and January were one category), 10-year age group, sex, presence of at least one of the commonly collected chronic conditions (except IE where "has any medical risk condition" is used).
[e] First dose of any COVID-19 vaccine received ≥14 days before onset.
[f] One of two recommended doses of COVID-19 vaccine received ≥14 days before onset. Excludes Janssen vaccine.
[g] Second dose of COVID-19 vaccine received ≥14 days before onset, or first dose received ≥14 days before onset if Janssen vaccine.

Supplementary Table S5. Odds ratio of COVID-19 vaccination by time since vaccination to symptom onset among participants receiving one dose only of 2-dose COVID-19 vaccines in the primary care/outpatient I-MOVE-COVID-19 VE study, Europe, December 2020–May 2021

**Secondary analysis (8 study sites[a])**				
**Analysis type and vaccination status**	**Cases / controls**	**Odds ratio (95% CI)[b]**	**Adjusted odds ratio (95% CI)[c]**	**Adjusted odds ratio (95% CI)[d]**
**Any COVID-19 vaccine**				
Unvaccinated	475/2214	Ref	Ref	
1–4 days[e]	30/207	0.86 (0.56 to 1.32)	0.83 (0.54 to 1.28)	0.83 (0.54 to 1.28)
5–13 days[f]	55/430	0.68 (0.49 to 0.95)	0.66 (0.47 to 0.92)	0.66 (0.47 to 0.93)
≥14 days[g]	40/482	0.44 (0.30 to 0.66)	0.44 (0.29 to 0.66)	0.44 (0.30 to 0.66)
**Comirnaty vaccine**				
Unvaccinated	473/2208	Ref	Ref	
1–4 days[e]	25/159	0.88 (0.55 to 1.40)	0.84 (0.52 to 1.34)	0.84 (0.53 to 1.35)
5–13 days[f]	48/341	0.71 (0.50 to 1.01)	0.68 (0.47 to 0.98)	0.69 (0.48 to 0.99)
≥14 days[g]	28/303	0.52 (0.33 to 0.81)	0.51 (0.32 to 0.82)	0.52 (0.33 to 0.84)
**Vaxzevria vaccine**				
Sample size too small				

CI: confidence interval.

[a] The secondary analysis excludes Scotland, a study site that is exclusively community-based and includes self-swabbing.
[b] Adjusted by study site, month of swab (due to low numbers December and January were one category).
[c] Adjusted by study site, month of swab (due to low numbers December and January were one category), 10-year age group, sex.
[d] Adjusted by study site, month of swab (due to low numbers December and January were one category), 10-year age group, sex, presence of at least one of the commonly collected chronic conditions (except IE where "has any medical risk condition" is used).
[d] First dose of 2-dose COVID-19 vaccine received 1–4 days before onset, patients with unknown doses or two doses excluded.
[f] First dose of 2-dose COVID-19 vaccine received 5–13 days before onset, patients with unknown doses or two doses excluded.
[g] First dose of 2-dose COVID-19 vaccine received ≥14 days before onset.

Sensitivity analyses

Supplementary Table S6. Sensitivity analyses of effectiveness of at least one dose of COVID-19 vaccine among participants in the primary care/outpatient I-MOVE-COVID-19 VE study, Europe, December 2020–May 2021

Analysis type and vaccination status	Cases; vaccinated/ controls; vaccinated	Adjusted VE (95% CI)[c]
Excluding FR	478;46 / 3164;861	64 (46-76)
Excluding IE	556;52 / 3149;862	62 (44-74)
Excluding NA	215;24 / 765;429	66 (38-82)
Excluding PT	559;52 / 3211;862	62 (45-74)
Excluding FR, IE, NA, PT	128;18 / 617;416	61 (17-81)
Imputing onset date as 2 days before the swab date	562;56 / 3218;885	60 (43 to 72)
Imputing onset date as 5 days before the swab date	566;46 / 3301;847	65 (48 to 77)
Data up to the end of April 2021	511;39 / 2735;574	62 (41 to 75)

CI: confidence interval; VE: vaccine effectiveness.
